# Cost estimate of hospital stays for premature newborns of adolescent mothers in a Brazilian public hospital

**DOI:** 10.1590/S1679-45082014GS2959

**Published:** 2014

**Authors:** Lutufyo Witson Mwamakamba, Paola Zucchi

**Affiliations:** 1 Universidade Federal de São Paulo, São Paulo, SP, Brazil

**Keywords:** Cost and cost analysis, Pregnancy in adolescence, Obstetric labor, premature, Infant, premature, Intensive care, neonatal

## Abstract

**Objective:**

To estimate the direct costs of hospital stay for premature newborns of adolescent mothers, in a public hospital.

**Methods:**

A cost estimate study conducted between 2009 and 2011, in which direct hospital costs were estimated for premature newborns of adolescent mothers, with 22 to 36 6/7 gestational weeks, and treated at the neonatal unit of the hospital.

**Results:**

In 2006, there were 5,180 deliveries at this hospital, and 17.8% (922) were newborns of adolescent mothers, of which 19.63% (181) were admitted to the neonatal unit. Out of the 181 neonates, 58% (105) were premature and 80% (84) of them were included in this study. These 84 neonates had a total of 1,633 days in-patient hospital care at a total cost of US$195,609.00. Approximately 72% of this total cost (US$141,323.00) accounted for hospital services. The mean daily costs ranged from US$97.00 to US$157.00.

**Conclusion:**

This study demonstrated that the average cost of premature newborns from adolescent mothers was US$2,328.00 and varied according to birth weight. For those weighing <1,000g at birth, the mean direct cost was US$8,930.00 per stay as opposed to a cost of US$642.00 for those with birth weight >2,000g. The overall estimated direct cost for the 84 neonates in the study totaled US$195,609.00.

## RESUMO

**Objetivo:**

Estimar os custos diretos de internações de recém-nascidos prematuros de mães adolescentes em um hospital público.

**Métodos:**

Estudo de estimativa de custos realizado entre 2009 e 2011, no qual os custos hospitalares diretos foram estimados para recém-nascidos prematuros de mães adolescentes, com 22 a 36 6/7 semanas de gestação, e tratados na unidade neonatal do hospital.

**Resultados:**

Em 2006, o hospital em estudo teve 5.180 partos; 17,8% (922) foram de mães adolescentes, e 19,63% (181) recém-nascidos foram admitidos na unidade neonatal. Dos 181 neonatos, 58% (105) eram prematuros, dos quais 80% (84) foram incluídos neste estudo. Estes 84 recém-nascidos totalizaram 1.633 dias de tratamento hospitalar, a um custo total de US$195,609.00. Aproximadamente 72% desse custo total (US$141,323.00) referiu-se a serviços hospitalares. Os custos médios diários variaram de US$97.00 a US$157.00.

**Conclusão:**

Este estudo demonstrou que o custo médio de recém-nascido prematuro de mães adolescentes foi US$2,328.00 e variou de acordo com o peso ao nascimento. Para recém-nascidos com peso <1.000g ao nascer, o custo médio foi US$8,930.00, e para os que pesaram >2.000g foi de US$642.00. O custo hospitalar total estimado para os 84 neonatos no estudo foi de US$195,609.00.

## INTRODUCTION

Since the 1980's, the number of newborn intensive care units (ICU) has increased dramatically. This increase was associated with significant costs and a considerable amount of heterogeneity between countries, or even within a country, when it comes to allocation of resources and distribution of critical care services.^(1)^

It is evident that there is a paucity of studies that specifically address intensive care costs,^(2)^ with variations in study populations and differing economic evaluation methods.^(3)^ Thus, there is constant need for cost evaluation and economic analyses especially about direct medical costs, which impact on the finances of the hospital.

Adolescent pregnancy, in which the mother is aged between 10 and 19 years, has become a multifaceted matter involving social, cultural, educational, health and economic issues of vast diversity. Consequently, the high cost of caring for neonates has raised much concern about preterm labor in adolescent mothers.^(4)^

The prevalence of preterm births varies from region to region. According to the World Health Organization, as of 2006 in the United States about 12 to 13% of all births were preterm.^(5, 6)^ These premature newborns (PN) accounted for expensive hospital treatments and numerous days of hospital care when compared to term infants.^(6, 7)^

### The Brazilian perspective

In Brazil, in 2006, 20% of the 186,770,613 inhabitants (38,822,165) were adolescents aged 10 to 19 years. Of these, 49% (19,251,110) were female according to the Brazilian Institute of Geography and Statistics (IBGE).^(8)^ Of the 2,944,928 neonates born in Brazil in 2006, 21% were born to adolescent mothers (27,610 mothers aged 10 to 14 years and 605,270 aged 15 to 19 years). Astonishingly, of the total number of 161,271 PN, 27.84% (44,906) were born to adolescent mothers, who had a preterm rate of 7%, compared to 5% among non-adolescents.^(9)^ However, it is important to note the variation of obstetrical characteristics of these pregnancies from one region to another, due to heterogeneous socioeconomic factors.^(10, 11)^ In terms of neonatal beds, in the same year of 2006, there was 1.6 neonatal bed per 1,000 inhabitants with the majority of these beds located in the city of São Paulo, both within the private and public sectors.^(12)^

## OBJECTIVE

To estimate direct hospital costs of premature newborns of adolescent mothers, in a public hospital.

## METHODS

### Study design and setting

A cost estimation study, conducted at *Centro Paulista de Economia da Saúde*, at the *Universidade Federal de São Paulo* (UNIFESP), at São Paulo (SP), Brazil, from 2009 to 2011. Data were collected from *Hospital Municipal e Maternidade-Escola Vila Nova Cachoeirinha Dr. Mario de Moraes Altenfelder Silva* (HMMEVNC), a tertiary public hospital in the city of São Paulo, located in one of the 96 districts, in the Northern region.

This hospital is classified as a healthcare facility level 8 by the Brazilian National Register of Healthcare Facilities (CNES, abbreviation in Portuguese) and provides high complex procedures to inpatients and outpatients.^(13^) The organization provides continuous service, 24 hours a day, 7 days per week, and it is a reference center for high-risk pregnant women and newborns of 18 districts.

The hospital had 170 planned beds and 155 operational beds, of which 52 were neonatal beds; in that, 20 were in ICU, 20 in the stepdown unit (SD) and 12 in intermediate care (IC). There were approximately 500 births per month.

The hospital team comprised the following full-time healthcare professionals: 84 nurse technicians, 32 neonatologists, 12 registered nurses, 7 physical therapists, and 2 speech therapists, as well as on-call professionals including, radiologists, cardiologists, neurosurgeons etc. The overall mean occupancy rate of the neonatal unit, calculated through daily census in 2006, was 75%, with an occupancy rate in each unit of 88% in ICU, 63% in SD and 80% in IC, and a mortality rate of 7%.

### Study population

The study included all adolescents aged 10 to 19 years, with singleton pregnancies delivered at the hospital, from January to December of the same year (2006), with preterm gestations of 22 to 36 6\7 weeks. Gestations of less than 22 weeks were not considered due to their unviability.^(14)^

It is evident that an accurate estimation of gestational age is paramount in obstetric care decisions that directly influence the PN. Obtaining an accurate and reliable gestational age in adolescents is influenced by numerous factors: the uncertainty regarding the last menses period and late onset of prenatal care, which alter the gestational age based on the ultrasound calculation.

In this study, the gestational age was determined based on clinical examination using the New Ballard score: a physical and neurological examination, performed in the first 12 hours of life by neonatologists. This test was considered due to the fact that there is a predictable pattern of changes occurring throughout gestation, and this is an accurate test when compared to other tests.^(15^)

The inclusion criteria were PN ≥24 hours of life, born at the HMMEVNC, admitted to the neonatal unit. The neonates were classified into four subgroups due to the level of risk and mortality that is directly related to weight (<1,000g, 1,000 to 1,499g, 1,500 to 1,999g and >2,000g).

The exclusion criteria were PN with severe congenital malformations or those that were transferred to other facilities for further treatments, as these factors made it difficult in obtaining reliable medical costs, as well as the impact on cost minimization or maximization depending on the severity.^(16)^ The PN born to mothers with a prior history of other medical conditions were also excluded in order to minimize the intrinsic adverse effects of these pathologies on pregnancy.

### Sources of data

The medical records were collected from the Medical Files Storage and Statistical Service of the hospital. Data on gestation, mothers and neonates were retrieved from the medical records and the newborn ICU.

Direct hospital costs, as used in this study, refer to “cost for goods and services used in diagnosis, prevention, treatment and rehabilitation of a patient”.^(17)^ Data relating to direct medical costs estimate were obtained from the hospital pharmacy, human resources, finance, central storage and purchasing departments and the neonatal unit. The costs were calculated from the day of birth to the day of discharge. The direct medical costs focused exclusively on care rendered in the NICU.

The direct cost of hospital care included the hospital services described below.

### Hospital services

High cost medications, such as surfactant, prostaglandins and others, have proven to have a significant impact on cost.^(18)^ The cost estimate for these drugs was based on the unit price of purchase at the time of utilization and multiplied according to individual usage. The cost of low cost medications, such as antibiotics, analgesic drugs and others, was based on the purchase price after summing up each medication in use by the neonatal unit, in 2006, divided by 365 days, and multiplied individually according to the utilization based on diagnosis and days of use. This method was also utilized for nursing care materials, such as syringes, cotton, assepsis products, and other, regardless of diagnosis.

The estimate of parenteral nutrition cost was based on daily purchase price and multiplied according to days of utilization.

Concerning medical gases, the gas and dispensing equipment were considered. For gases such as oxygen, the cost estimate was done after calculating the cost per cubic meter of oxygen delivered at 1L/minute. An average of oxygen utilization per L/minute was determined in three modalities (orotracheal ventilation/nasal ventilation and oxygen therapy without invasive procedures). The average obtained was further multiplied according to days of utilization in each modality by the patient. This value was added to the ventilating equipment when applicable, and the cost estimate was calculated by taking into account purchase and maintenance values, accumulated depreciation and utilities necessary to main the equipment.^(19)^ This same method was applied to estimate the cost of other pieces of equipment, such as monitors, and estimates were calculated according to days of use by each patient.

Clinical services were defined as support services that are directly related to patient care, but were not supplied by the ICU, such as radiology, nutrition, speech therapy and audiology, physical therapy etc. In this study, two procedures were considered; physical therapy and speech therapy and audiology. The cost estimate was based on the number of sessions multiplied by days of utilization, and the values were obtained from the List of Medical Procedures published by the Brazilian Medical Association (abbreviation in AMB).^(20)^ The other procedures were not included due to incomplete financial information that could be tracked directly to the patient.

Manpower was defined as the net pay for nursing and medical staff employed full-time in the ICU. The cost of nursing services was determined on the basis of the average salary of nurses who had been employed for an average of 2 years. The total number of nurse-hours was calculated for the sector, and the total of all tallies was divided by this total number of hours to obtain the cost per hour worked within this category. Medical services were based on the average salary of neonatologists who had worked for 2 years at the hospital. The salaries were calculated in full and calculated according to working hours.

Diagnostic tests were additional exams to help making clinical diagnoses, as blood tests, biochemical profiles, blood gas analysis, immunology tests, X-rays, ultrasounds, and eye exams. The costs of these exams were obtained from the AMB list.^(20)^

Costs of blood products were calculated according to the tables available from private blood banks for red blood cell concentrate, plasma, platelets and coagulation factors.

The costs related to electrical power, water, telephone services, administrative expenses, general expenses, cleaning, maintenance, laundering, diet and nutritional expenses were considered as indirect costs that comprise the hospital overhead and were not included in the study. The results of this study were compared with those of international studies by exchanging the values of this study from Brazilian currency (*real*) into US dollars, at an exchange rate of 2.25354 (the annual average rate in 2006).^(21)^

### Ethical aspects

Ethical approval for this study was obtained from the ethical and research committees of *Maternidade Municipal Escola de Vila Nova Cachoeirinha*, protocol 011/2007, and of *Escola Paulista de Medicina of Universidade Federal de São Paulo*, protocol 1961/07.

### Statement of consent

The objective of this study is to estimate direct hospital costs of premature newborns of adolescent mothers, in a public hospital in the city of São Paulo (SP), Brazil.

Data collection was conducted by the investigator using patient's medical records and other resources without any risk to the patients or to the organization of the study, with data exclusive for this study. At any stage of the project, the data will be available for any clarification. It assures freedom to withdraw consent at any time and termination of this study without any prejudice to the institution and constant updating. It also assures the right of confidentiality of patients' information, with no disclosure of their identification.

## RESULTS

In 2006, the hospital had 5,180 newborns with more than 24 hours of life. Of these newborns, 17.8% (922) were from adolescents, of which 19.63% (181) were admitted to the neonatal unit. Of the 181 neonates admitted to the neonatal unit, 58% (105) were PN and 80% (84) were included in this study. The remaining 20%, 8% were younger than 24 hours of life, 7% had congenital malformations and the others could not be included due to incomplete medical records. The 84 PN were classified as follows: 4 neonates <1,000g; 13 neonates with 1,000 to 1,499g; 32 neonates with 1,500 to 1,999g; 35 neonates with 2,000 to 2,499g.

The mean age observed in the 84 adolescent mothers was 16 years, range of 14 to 19 years, about 58.33% (49) were single mothers, 84.52% (71) had completed the 8th grade (junior school), while 71.42% (60) were primigravid and 79.76% (67) had at least three prenatal care appointments.

Among the 84 adolescents, 48 hours prior to birth: 47.62% (40) received treatments as inpatients, including tocolytics, corticosteroids, antibiotics and rest; 35.71% (30) received treatments as outpatients, which consisted of antibiotics and rest, and 14% (17) had no specific treatments.

As to the probable cause of preterm labor, the following diagnosis at the onset of preterm labor were identified: urinary tract infection in 60.0% (50) of these adolescents; gestational hypertension in 11.90% (10); preterm labor of unknown cause in 11.90% (10); premature rupture of membranes, onset of labor within 24 hours in 11.90% (10); and abruption placentae in 4 (4.28%).

### Costs

Tables 1 to 4 illustrate the calculated direct hospital costs of the PN according to the subgroups and variables used in direct hospital cost estimate, including hospital services, diagnostic tests and blood products as well as length of stay accumulated by these PN.

**Table 1:** Distribution of hospital stay according to birth weight

Birth weight		<1,000g	1,000-1,499g	1,500-1,999g	>2,000g
NB, n		4	13	32	35
Total days of hospital care		227	637	539	230
	Average	56.75	49.0	16.80	6.57
Length of stay at ICU (days)		129	339	190	55
	Average	32.25	26.0	5.93	1.57
Length of stay at SD (days)		98	298	300	26
	Average	24.5	22.90	9.34	0.74
Length of stay at IC (days)		–	–	49	149
	Average	–	–	1.53	4.25

NB: newborn; ICU: intensive care unit; SD: step-down unit; IC: intermediate care unit.

**Table 2:** Cost of hospital services for premature newborns by birth weight

Birth weight		<1,000g	1,000-1,499g	1,500-1,999g	>2,000g
NB, n		4	13	32	35
Hospital services					
	Medicinal gases: total days of use O_2_	118	305	162	56
	Total cost of O_2_ utilization	US$3,045.00	US$4,755.00	US$1,905.00	US$627.00
	Cost O_2_ per NB	US$759.00	US$366.00	US$60.00	US$18.00
	Parenteral nutrition utilization, number of requests	49	83	35	12
	Total cost of parenteral nutrition per NB	US$2,500.00	US$4,194.00	US$1,775.00	US$612.00
	Average cost of parenteral nutrition per NB	US$625.00	US$326.00	US$56.00	US$26.00
	Nursing supplies per NB	US$917.00	US$814.00	US$229.00	US$180.00
	Total value of nursing materials	US$3,668.00	US$10,582.00	US$7,328.00	US$6,300.00
	Average cost of low cost medications per NB	US$887.00	US$819.00	US$190.00	US$40.00
	Total costs of low cost medications	US$3,548.00	US$10,647.00	US$6,080.00	US$1,398.00
	Average cost of high cost medications per NB	US$641.00	US$232.00	US$47.00	US$7.00
	Total costs of high cost medications	US$2,564.00	US$3,016.00	US$1,504.00	US$245.00
	Special material per NB	US$117.25	US$6,769.00	US$625.00	US$485.00
	Total value of special supplies	US$469.00	US$880.00	US$200.00	US$170.00
	Professional services: Average cost of professional services per NB	US$2,336.00	US$2,022.00	US$671.00	US$183.00
	Total costs of professional services	US$9,353.00	US$26,015.00	US$21,530.00	US$6,404.00
	Total costs of hospital services	US$25,146.00	US$60,089.00	US$40,322.00	US$15,756.00
	Average cost of hospital services per NB	US$6,286.00	US$4,622.00	US$1,260.00	US$450.00

O_2_: oxygen; NB: newborn

**Table 3:** Proportional composition of hospital services, diagnostic tests and blood products by weight

Birth weight		<1,000g	1,000-1,499g	1,500-1,999g	>2,000g
Number of newborns		4	13	32	35
Professional services					
	Physicians	US$2,545.00	US$7,135.00	US$5,787.00	US$1,946.00
	Nursing staff	US$6,054.00	US$16,054.00	US$13,391.00	US$3,614.00
	Speech therapists and physical therapists	US$754.00	US$2,826.00	US$2,352.00	US$834.00
	Total costs of professional services	US$9,353.00	US$26,015.00	US$21,530.00	US$6,404.00
	Average cost of professional services per NB	US$2,338.00	US$2,001.00	US$673.00	US$183.00
Diagnostic tests					
	Radiology	US$2,451.00	US$4,430.00	US$3,507.00	US$1,469.00
	Hemotological tests	US$7,292.00	US$15,297.00	US$12,081.00	US$5,158.00
	Microbiology	US$378.00	US$515.00	US$755.00	US$644.00
	Average costs per NB	US$2,530.00	US$1,557.00	US$510.00	US$189.00
	Total costs of diagnostic tests	US$10,121.00	US$20,242.00	US$16,343.00	US$6,627.00
Blood products					
	Total costs of blood products: red blood cells, plasma, platelets concentrate	US$455.00	US$411.00	US$142.00	US$97.00

NB: newborn.

**Table 4:** Direct costs of hospital stay for preterm newborns in the neonatal intensive care unit

Birth weight	<1,000g	1,000-1,499g	1,500-1,999g	>2,000g
Number of newborns	39	13	32	35
Total length of stay, days	227	637	539	230
Average length of stay, days	56	49	16.8	6.6
Hospital services	US$25,146.00	US$60,089.00	US$40.322.00	US$15,756.00
Diagnostic tests	US$10,121.00	US$ 20,242.00	US$ 16,343.00	US$6,627.00
Blood products	US$455.00	US$411.00	US$142.00	US$97.00
Overall total costs	US$35,722.00	US$80,742.00	US$56,665.00	US$22,480.00
Average cost per NB	US$8,930.00	US$6,210.00	US$1,770.00	US$642.00
Daily cost in the NU	US$157.00	US$126.00	US$105.00	US$97.00

NB: newborn; NU: neonatal unit.

## DISCUSSION

### Brazilian perspective

In 2006, Brazil had a population of 19 million female adolescents. The main reason for admission to hospital of 72% of this population was pregnancy and labor-related conditions. The probability of delivering PN was 16 to 31% as compared to 7 to 8.65% of non-adolescent mothers.^(8, 22)^

In this study, carried out with PN from adolescent mothers with singleton pregnancies, we evidenced that the average direct cost of hospital stays for 84 PN was US$2,328.68 and the total cost was US$195,609.00.

Although admissions for PN weighing <1,000g accounted for only 4.76% of all preterm admissions, this group incurred approximately 18.26% of the total PN direct hospital costs. In addition, PN of 1,000g to 1,999g accounted for 58% of all preterm admissions, and they accounted for approximately 70% of the total direct costs. Finally, PN >2,000g represented 41.66% of the 84 PN admitted to the neonatal unit, incurring about 10% of hospital costs.

The median length of stay for all 84 PN was 19.44 days; hospital services accounted for 72.22% of all costs, of which 44.80% dedicated to salaries, and 27.26% spent on therapeutic and diagnostic services. PN weighing ≤1,000g accounted for average costs per newborn of US$8,930.00, and neonates weighing >2,000g, incurred the lowest cost (US$642.00).

These results are consistent with numerous other studies, regardless heterogeneous sources, designs and methods, that is, 45 to 75% of direct neonatal costs were due to salaries, and an overall significant hospital cost varied according to birth weight.^(23^)

Comparing this study to other investigations conducted in Brazil, we noted that there are scarce studies on direct hospital costs, specifically on PN of adolescent mothers. Study conducted in a public hospital in the city of Sao Paulo, in 2004, estimated the direct hospital costs of PN of all pregnant women. Authors indicated cost variation, as neonates weighing <1,000g and from 1,000 to 1,499g had mean hospital stay of 34 and 42 days, with average neonatal costs of US$3,919.00 and US$4,043.00, respectively. For those weighing from 1,500 to 1,999g and >2,000 g, the average cost ranged from US$1,768.00 to US$1,183.00, and the total direct cost of treatment for PN was US$2,017.00.^(24)^

This study showed that PN weighing <1,000g and from 1,000 to 1,499g had a mean hospital stay of 56 and 49 days, with average neonatal costs of US$8,930.00 and US$6,210.00, respectively. The difference in costs may be attributed to comorbidities that are associated with PN in adolescents that are directly proportional to costs, such as prenatal care.^(25, 26)^ Thus there is potential risk of developing several conditions that is inversely proportional to vulnerability of gestational age.^(27^)

Based on the Ministry of Health estimates of costs per diagnosis, in 2006, the average cost of PN was US$979.00 *versus* US$223.65, in general pediatric care.^(28)^ These values further demonstrate the underestimated data from the government in terms of costs.

### Limitations of the study

It was necessary to round up calculations due to lack of an accounting system that evaluates costs of hospital stay. Such system is not always available, especially in public organizations in Brazil.^(18^) Besides, ancillary costs were not included in this study, and they may sum up to 15.7% of overall overhead costs.^(29)^

Despite the fact that this study was conducted in 2011, with data available from 2006, it can be observed that the Brazilian accumulated annual inflation rate (IPCA, Índice Nacional de Preços ao Consumidor Amplo, in Portuguese), from 2006 to 2012, amounts to 36.03% of costs.^(9)^ The total direct cost of US$195,609.00, in 2006, would be US$266,028.00, in 2012.^(30)^

Finally, the reference of payments by the Brazilian public health system and the fees established by the AMB to officially calculate costs are extremely outdated, leading to a potential risk of cost underestimate.^(13, 20)^

## CONCLUSION

Despite the general notion that premature newborn leads to high medical cost, in Brazil there are scarce studies estimating the direct medical neonatal costs for these babies, specifically those of adolescent mothers. This study was conducted a public hospital and demonstrated that the average cost of premature newborn of adolescent mother was US$2,328.00. The costs varied according to birth weight and decreased exponentially with advanced gestational age, whereas premature newborn weighing <1,000g had an average direct cost of US$8.930,00 *versus* US$642.00 for those weighing >2,000g. The overall estimated direct cost with 84 neonates totalled up US$195,609.00
